# Bifidobacteria exhibit social behavior through carbohydrate resource sharing in the gut

**DOI:** 10.1038/srep15782

**Published:** 2015-10-28

**Authors:** Christian Milani, Gabriele Andrea Lugli, Sabrina Duranti, Francesca Turroni, Leonardo Mancabelli, Chiara Ferrario, Marta Mangifesta, Arancha Hevia, Alice Viappiani, Matthias Scholz, Stefania Arioli, Borja Sanchez, Jonathan Lane, Doyle V. Ward, Rita Hickey, Diego Mora, Nicola Segata, Abelardo Margolles, Douwe van Sinderen, Marco Ventura

**Affiliations:** 1 Laboratory of Probiogenomics, Department of Life Sciences, University of Parma, Italy; 2 APC Microbiome Institute and School of Microbiology, Bioscience Institute, National University of Ireland, Cork, Ireland; 3 Departamento de Microbiologia y Bioquimica de Productos Lacteos, IPLA – CSIC, Villaviciosa, Asturias, Spain; 4 Centre for Integrative Biology, University of Trento, Trento, Italy; 5 Teagasc Food Research Centre, Moorepark, Cork, Ireland; 6 Broad Institute, 415 Main St., Cambridge, USA; 7 Department of Food Environmental and Nutritional Sciences, University of Milan, Italy

## Abstract

Bifidobacteria are common and frequently dominant members of the gut microbiota of many animals, including mammals and insects. Carbohydrates are considered key carbon sources for the gut microbiota, imposing strong selective pressure on the complex microbial consortium of the gut. Despite its importance, the genetic traits that facilitate carbohydrate utilization by gut microbiota members are still poorly characterized. Here, genome analyses of 47 representative *Bifidobacterium* (sub)species revealed the genes predicted to be required for the degradation and internalization of a wide range of carbohydrates, outnumbering those found in many other gut microbiota members. The glycan-degrading abilities of bifidobacteria are believed to reflect available carbon sources in the mammalian gut. Furthermore, transcriptome profiling of bifidobacterial genomes supported the involvement of various chromosomal loci in glycan metabolism. The widespread occurrence of bifidobacterial saccharolytic features is in line with metagenomic and metatranscriptomic datasets obtained from human adult/infant faecal samples, thereby supporting the notion that bifidobacteria expand the human glycobiome. This study also underscores the hypothesis of saccharidic resource sharing among bifidobacteria through species-specific metabolic specialization and cross feeding, thereby forging trophic relationships between members of the gut microbiota.

Bifidobacteria are Gram positive bacteria with high GC-content genomes that typically reside in the gastro intestinal (GIT) tract of various animal species, including warm blooded mammals and social insects^1^,^2^. Noteworthy, bifidobacteria reach a very high relative abundance as part of the infant gut microbiota^3^,^4^,^5^, and this early life prevalence supports their purported role as modulators of various metabolic and immune activities of their host^1^. Non-digestible carbohydrates derived from the diet, together with host-produced glycans found in the mammalian gut represent critical energy sources believed to be responsible for the survival and proliferation of many microbial components of the gut microbiota, including bifidobacteria^6^. It is therefore important to know what molecular strategies are employed by members of the gut microbiota to harvest and metabolize complex glycans in order to understand how they underpin their ecological fitness in, and adaptation to, the GIT environment. Furthermore, carbohydrate metabolism in the mammalian gut may occur in a concerted manner by various members of the microbiota, including bifidobacteria^7^. Thus, carbohydrates are presumed to be partly responsible for microbiota dynamics in this changeable environment. Genomics has been crucial in revealing the complex interactions and molecular dialogue between a host and its resident (bifido)bacteria, and in unraveling bifidobacterial gut colonization strategies^8^. Recently, all 47 currently recognized bifidobacterial sub/species have been genomically decoded^9^,^10^, thus providing the necessary genetic background for this group of bacteria. Here, we describe an analysis of the genomes and transcriptomes of representatives of all 47 (sub)species that are currently assigned to the *Bifidobacterium* genus, predicting bifidobacterial genome-based strategies for carbohydrate metabolism that impact on the overall glycobiome of the gut microbiota and host, while also being pivotal for the establishment of trophic relationships between members of gut microbiota.

## Results and Discussion

### Genomes of the *Bifidobacterium* genus and carbohydrate metabolism

Genome sequences from the type strain of each of the 47 *Bifidobacterium* (sub)species (Table S1) were used to assess the contribution of carbohydrate metabolism to the corresponding pan-genome, core genome and variome, determined as described previously^9^. In total, 18,181 and 551 BifCOGs (*Bifidobacterium*-specific Clusters of Orthologous Genes) represent the pan-genome (pan BifCOGs) and the core of bifidobacterial genomic coding sequences (core BifCOGs) of the *Bifidobacterium* genus, respectively. Functional annotation of the core BifCOGs, based on a recently updated EggNog database^11^, indicates, as expected, that a large part of the conserved core genes encode housekeeping functions and functions involved in the adaptation to/interaction with a particular environment, including carbohydrate metabolism (Fig. S1). Interestingly, about 5.5% of the core BifCOGs is associated with carbohydrate metabolism (Fig. S1), whereas the carbohydrate metabolism functional family is the most represented COG family of the pan BifCOGs (13.7%) (Fig. S1). This finding suggests a strong selective pressure towards the acquisition and retention of accessory genes for carbohydrate utilization by bifidobacteria in order to be competitive in a particular ecological niche. The commitment of bifidobacteria towards the utilization of a wide array of simple and complex carbohydrates is further underlined by EggNog profiling of adult human’s faecal metagenomes sequenced as part of the Human Microbiome Project (HMP). Results showed that the average abundance of the carbohydrate metabolism functional family in HMP metagenomic datasets is 8.0%, thus being 58% lower than the abundance detected in the bifidobacterial pan-genome, i.e. 13.7% (Fig. S1). Notably, the carbohydrate metabolism functional family is the most abundantly represented COG family within the *Bifidobacterium* pan-genome (Fig. S1). The pan-genome analysis also allowed the identification of the Truly Unique Genes (TUG), consisting of genes present in just one of the examined bifidobacterial genomes (Fig. S1). Interestingly, no functional annotation can be made for the majority of TUGs (54.1%) (Fig. S1). However, 13.2% of the identified TUG can be attributed to a COG family representing proteins involved in carbohydrate metabolism, including glycosyl hydrolases (GH) and carriers for carbohydrate uptake. This data supports the notion that carbohydrate metabolism supports adaptation and specialisation processes, and consequently speciation within the *Bifidobacterium* genus.

### Carbohydrate-active enzymes encoded by the genus *Bifidobacterium*

**Figure 1:**
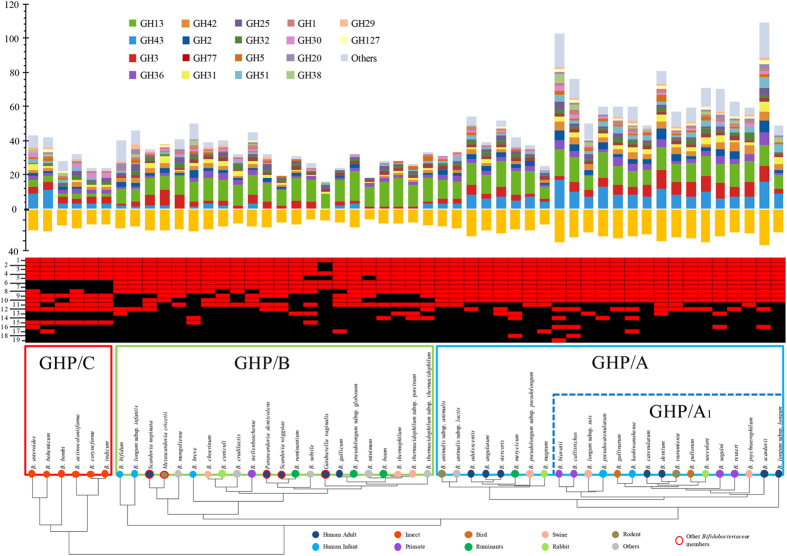
Predicted glycobiome of the *Bifidobacterium* genus and some additional members of the *Bifidobacteriaceae* family. GH families and carbohydrate-utilization pathways profiles, based on CAZy database and Pathway-tools software, respectively, were used to construct a hierarchical clustering of all tested species of the *Bifidobacterium* genus and additional members of the *Bifidobacteriaceae* family. This clustering highlights the presence of three distinct clusters named GHP/A, GHP/B and GHP/C that display a different repertoire of GHs as well as a different repertoire of plant carbohydrate degradation pathways. GH arsenal prediction for every analyzed *Bifidobacteriaceae* species is represented by a bar plot. The presence of pathways for degradation of simple or complex carbohydrates is represented by the red color in the heat map and the GH index (the number of GHs predicted in each genome normalized by genome size expressed as Mbp) is illustrated as an orange bar plot. Pathways denominations are indicated as follows: 1 *Bifidobacterium* shunt, 2 galactose degradation I (Leloir pathway), 3 melibiose degradation, 4 ribose degradation, 5 lactose degradation III, 6 glycogen degradation I, 7 glycogen degradation II, 8 sucrose degradation IV, 9 L-arabinose degradation I, 10 xylose degradation I, 11 D-mannose degradation, 12 (1,4)-ß-xylan degradation, 13 starch degradation V, 14 chitin degradation (chitinase), 15 trehalose degradation IV, 16 Pectin (homogalacturonan) degradation, 17 2'-deoxy-a-D-ribose 1-phosphate degradation, 18 trehalose degradation I (low osmolarity), 19 L-rhamnose degradation II.

Considering the importance of carbohydrate metabolism and energy conversion systems in gut inhabitants such as bifidobacteria, we decided to investigate the presence of genes predicted to encode GHs, polysaccharide lyases (PLs), carbohydrate esterases (CE), glycosyl transferases (GT) and carbohydrate binding modules (CBM), and other carbohydrate metabolic pathway components using the predicted core- and pan-genome of the *Bifidobacterium* genus. Genomic data showed that *B. scardovii* and *B. biavatii* have a significantly larger set of genes involved in carbohydrate metabolism, also termed glycobiome^12^, as compared to other bifidobacterial species (Fig. 1). Normalization of GH counts against genome size (Mbp), generating a GH index, provided an insight into the relative extent of adaptive events related to carbohydrate metabolism found in individual bifidobacterial species. Notably, four species (*B. scardovii, B. biavatii, Bifidobacterium saeculare* and *Bifidobacterium dentium*) possess a GH index that is approximately 50% higher than the bifidobacterial average (Fig. 1). Classification according to the Carbohydrate Active Enzymes (CAZy) system^13^ revealed that the pan-genome of the analysed *Bifidobacterium* representatives includes 3,385 genes encoding predicted carbohydrate-active enzymes, including members of 57 GH, 13 GT and seven CE families (Fig. 1, Fig. S2 and Fig. S3), while no putative Polysaccharide Lyases (PL)-encoding genes were detected. Genes encoding members of CAZy family GH13 are most commonly found in bifidobacterial genomes, especially for those bifidobacteria isolated from the mammalian gut (24.2% of the total predicted bifidobacterial GH repertoire, Fig. 1). Enzymes belonging to this family are widespread in bacteria and are characterized by their degradative abilities towards a wide range of carbohydrates, including plant-derived polysaccharides, such as starch and related substrates (amylose and amylopectin and/or (cyclo)maltodextrins), and trehalose. Furthermore, stachyose, raffinose and melibiose may also represent a target for members of the GH13 family^14^ and their complete breakdown is achieved with the involvement of GH36 enzymes, which were shown to be abundant in the analysed bifidobacterial genomes (Figure S2). Such carbon sources indeed represent very common glycans found in the adult mammalian (omnivore and herbivore) diet^6^. We also observed distinct differences between predicted glycobiomes of *Bifidobacterium* species with regards to their (predicted) ability to degrade plant polysaccharides as opposed to host-derived glycans, such as N- or O-linked glycoproteins, human milk oligosaccharides (HMOs) and glycosaminoglycans^1^. The bifidobacterial glycobiome was shown to contain members of GH families that are known to be involved in host-glycan degradation, such as GH33, encompassing exo-sialidases, GH29 and GH95, which represent fucosidases, GH20, which include hexosaminidase and lacto-N-biosidase activities, GH112, representing lacto-N-biosidases, GH38 and GH125, involving α-mannosidases as well as GH101 and GH129, which include α-N-acetylgalactosaminidases. Interestingly, members of the *B. scardovii*, *B. longum* subsp. *infantis* and *Bifidobacterium bifidum* species possess the most extensive set of such host-glycan-degrading GH families (Fig. 1). Such glycobiome specialization probably reflects species-specific adaptation to a particular ecological niche and illustrates strict co-evolution with their mammalian host. Clustering of bifidobacterial species based on their predicted GH and carbohydrate-degradation pathway repertoire (Fig. 1) allows the identification of a cluster, designated as GHP/A, representing species with a considerable array of predicted GH43 family enzymes involved in degradation of complex plant polysaccharides, such as (arabino)xylan, which plays an important structural role as a main constituent of the plant cell wall^15^, and as such represents a substantial component of plant cell wall-derived dietary fiber^16^. This finding suggests that bifidobacteria rich in GH43 member-specifyings genes are adapted to hosts with a vegetarian or omnivore diet. A subgroup of the cluster GHP/A, named GHP/A1, appears to possess a wider array of GHs (Fig. 1). This subgroup encodes a large number of GH3 family enzymes that are predicted to be involved in the degradation of an extensive range of plant-derived polysaccharides (e.g., cellodextrin, (arabino)xylan and (arabino)galactan), as well as involved in bacterial cell wall biosynthesis and turnover. Notably, bifidobacterial species isolated from honey/bumblebees specify a peculiar GHP/C cluster, whose members possess a discrete set of GH43 and GH3 enzymes, but in addition these particular bifidobacterial genomes are predicted to encode a very limited repertoire of GH13 representatives, in contrast to all other bifidobacteria. This is in accordance with the absence or paucity of carbohydrates with α-glucosidic linkages in the honey/bumblebee diet^17^. The remaining bifidobacterial species, not fitting in clusters GHP/A or GHP/C, are included in cluster GHP/B, which is characterized by an under-representation of GH43 and GH3.

**Figure 2:**
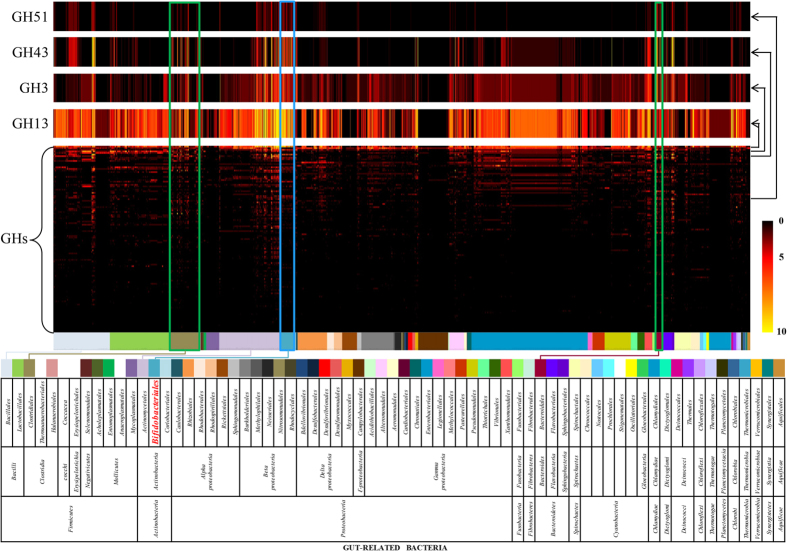
Comparative analysis of bifidobacterial GHs against other gut bacteria. The central heat map shows GH prediction data of 2721 sequenced bacterial strains belonging to bacterial orders residing in the human gut, identified by different color codes as explained in the underlying table. The four heat map rows, situated above the main heat map, represent an enlarged view of the GH51, GH3, GH43 and GH13 content. Data regarding *Bifidobacteriales* are highlighted in blue. Data regarding *Clostridiales* and *Bacteroidales* are highlighted in green.

In order to further evaluate the importance of the bifidobacterial contribution to degradation of complex carbohydrates found in the human gut, we compared the bifidobacterial GH repertoire with all the so far sequenced members of the human gut microbiome^18^,^19^. For this purpose, we used GH data of 2,674 genomes available in the CAZy database^13^ with the addition of data obtained from the bifidobacterial genomes analysed in this study. Interestingly, the *Bifidobacterium* genus was shown to specify one of the largest arsenal of GH13, GH43, GH3 and GH51 family members (2.0 fold-, 2.6 fold-, 5.8 fold-, 7.0 fold-, respectively, more with respect to the average GH arsenal of the gut microbiome), along with *Bacteroides* spp. (*Bacteroidales* family) and *Clostridiales* family. Furthermore, abundance of these GH families has been shown by a small number of other members of the gut microbiota such as *Paenibacillus* spp. (*Bacillales* family) and *Streptomyces* spp. (*Actinomycetales* family) (Fig. 2). These findings corroborate the very substantial contribution to glycan-breakdown potential by bifidobacteria in the mammalian gut.

### Secreted glycosyl hydrolases of bifidobacteria

In order to allow GH enzymes produced by different organisms to act in concert and to access glycans with a degree of polymerization exceeding that of the corresponding uptake system, certain GH enzymes are expected to be located on the cell surface or secreted into the environment. Notably, even though the majority of the identified GH enzymes from the bifidobacterial pan-genome is predicted to be intracellular, 10.9% of the deduced bifidobacterial GH pool is predicted to be extracellular, of which 32.9% are members of the GH13 family and annotated as pullulanases and α-amylases, 24% are members of the GH43 family and predicted to act as β-xylosidases and α-L-arabinofuranosidases, while 12% are members of the GH51 family and classified as α-L-arabinofuranosidases. These data suggest that bifidobacteria secrete a relevant pool of GH for the degradation of plant polysaccharides, which would accordingly represent an important resource for the host in the context of obtaining access to dietary fibres. The secreted bifidobacterial pan-genome also encompasses GHs involved in host-glycan degradation (see above), being classified as families GH29, GH33, GH95 and GH101, and constituting 0.4%, 3.5%, 0.8% and 2.7% of this secreted pan-genome, respectively. Predicted extracellular GHs were identified in 43 species of the genus *Bifidobacterium*, with a particularly high prevalence in *B. biavatii* (endowed with 17 secreted GHs, including 4 GH43 and 4 GH13 members), *B. scardovii* (endowed with 11 secreted GHs, including 3 GH43 and 3 GH51, annotated as α-L-arabinofuranosidases) and *B. bifidum* (endowed with 11 secreted GHs, including two GH84 and two GH33 members, predicted to act as N-acetyl β-glucosaminidases and sialidases, respectively). The remaining bifidobacterial genomes are predicted to encode seven or less secreted GHs. The two secreted N-acetyl β-glucosaminidases and the two secreted sialidases found in *B. bifidum* are crucial for the utilization of HMOs and intestinal glycoconjugates such as mucin, thus further supporting the notion that this microorganism is highly adaptated to colonize and persist in the mammalian gut^20^,^21^. Nevertheless, *B. bifidum* is unable to use sialic acid as its sole carbon source and the activity of sialidases appears to be important only to allow access to other carbohydrates associated to sialylated host-encoded glycans^20^,^21^. Furthermore, the released sialic acid can be utilized by other bifidobacteria, such as *B. breve*, resulting in cross-feeding between bifidobacterial species that share the same ecological niche^20^,^21^.

### Saccharolytic pathways of bifidobacteria

Predictions of complete pathways for the degradation of simple (di/trisaccharides) and complex sugars through Pathway Tools software^22^ showed that *B. biavatii* specifies the largest number of such pathways (14 complete pathways) (Fig. 1), while the predicted repertoire for carbohydrate degradation of *B. bombi, Bifidobacterium crudilactis, B. longum* subsp. *infantis, B. minimum* and *Bifidobacterium ruminantium* is limited to just nine pathways (Fig. 1). Notably, when assessing presence/absence data of pathways involved in the breakdown of simple and complex carbohydrates shown in the GHP clustering profile (Fig. 1), bifidobacterial species isolated from honey/bumblebees, constituting cluster GHP/C (Fig. 1), lack genes encoding glycogen degradation I and glycogen degradation II pathways. Based on recent discoveries, glycogen metabolic pathways are present in bacterial species able to face diverse environments and showing a flexible lifestyle^23^. More recently, it was reported that the ability of *Lactobacillus acidophilus* to synthesize and store energy in the form of glycogen, either prior to or during its transit through the host, potentially confers a competitive advantage in the GI tract^24^. This is supported by the fact that glycogen storage is ubiquitous among enteric bacteria, possibly due to the necessity to support rapid growth in the intestinal environment where there is intense trophic competition^25^. On the other hand, it was hypothesized^26^ that the loss of glycogen pathways is a strong indication of genome degradation associated with parasitic or symbiotic behaviour of bacteria. Though the original study of Henrissat *et al.* was based on the analysis and comparison of just 55 fully sequenced bacterial genomes, their findings have more recently been substantiated by others involving 1202 bacterial proteomes^23^. Among *Bifidobacteriaceae*, it is noteworthy that *B. actinocoloniiforme, B. asteroides, B. bohemicum, B. bombi, B. coryneforme* and *B. indicum*, species isolated from insects and constituting the GHP/C cluster, show a smaller genome size compared to all other bifidobacterial species isolated from mammals. We therefore speculate that *Bifidobacterium* members linked to insects have enjoyed a longer adaptation history to their hosts, in evolutionary terms, because mammals appeared on earth in the late Paleocene (between 65 and 23 mya), whereas insects in the Devonian period (between 459 and 359 mya) or even before according to a recent phylogenomics study^27^. This hypothesis is confirmed by comparison of the phylogenetic supertree of all known bifidobacterial species and that obtained for their corresponding hosts, revealing a co-evolution host-microbe profile (Fig. S4).

The genomes of *B. asteroides*, *B. actinocoloniiforme, B. indicum, B. coryneforme, B. bombi* and *B. bohemicum* possess a complete trehalose degradation IV pathway, which is absent in the majority of the other bifidobacteria and in all other examined members of the family *Bifidobacteriaceae*. The acquisition of this pathway, which is apparently specific for bifidobacterial species isolated from the insect gut, may be related to the fact that trehalose is used as carbohydrate storage and blood-sugar by many insects including bees^28^. Remarkably, a large majority of bifidobacterial genomes included in cluster GHP/B do not encompass pathways for L-arabinose and/or xylose metabolism (Fig. 1). These genomes encode relatively few GH43 enzymes compared to the GHP/A and GHP/C clusters, thus confirming a strict genetic adaptation to ecological niches where these plant carbohydrates are not available. The ecological origin of most of the GHP/B members that do not possess L-arabinose and/or xylose degradation pathways is either raw/fermented milk or faecal material/gastrointestinal tract of suckling animals (Table S1), where a milk-based diet represents the main nutrient retrieval opportunity. In this context, *B. breve, B. bifidum* and *B. longum* subsp. *infantis* have been isolated from infants, *B. thermacidophilum* subsp. *porcinum* and *B. choerinum* from piglet, *B. crudilactis* from raw bovine milk and *B. mongoliense* from fermented mare’s milk (Table S1), thus suggesting that these species have evolved to focus solely or predominantly on the degradation of carbon sources present in milk and have lost or did not acquire the ability to use (certain) plant polysaccharides. Fermented milk is a strict anthropogenic environment and these bacteria are unlikely to have evolved in fermented milk *per se*. In fact, the natural ecological environment of these species is still expected to be the gut from various (suckling) animals. In this environment such bifidobacterial taxa have enjoyed the presence of a rich reservoir of milk-based carbohydrates (oligosaccharides/lactose), which thus caused their genomes to acquire a genetic arsenal that allowed them to access these carbon sources. In contrast, *B. ruminantium* and *B. boum* taxa have been isolated from the bovine rumen (Table S1), an environment rich in plant polysaccharides, although they lack GH43-encoding genes and consequently cluster in GHP/B. Other rumen-derived bifidobacteria do encode GH43 enzymes, perhaps indicating that certain bifidobacteria rely on cross-feeding, which is in line with bifidobacteria being a minor component of the rumen microbiota, although their functional role is largely unknown. *B. breve* deserves a special mention as this species seems to have adopted a non-specialist strategy of acquiring constituent elements of both plant- and host-derived carbohydrates (consistent with its isolation from both infants and adults), yet is lacking the ability to directly access (many) HMOs, or mucin, nor possessing the ability to metabolize xylose/arabinose-containing carbohydrates, though it can metabolize a wide range of α/β-glucose- and α/β-galactose-containing sugars^14^,^20^. Thus, it may, perhaps co-operatively, rely on other (bifido)bacterial species like *B. bifidum* or *B. longum* subsp. *infantis* in order to sustain growth on the above mentioned complex sugars^29^.

### Carbohydrate utilization patterns of *Bifidobacterium*

Fermentation profiles of the 47 sequenced *Bifidobacterium* strains revealed that all strains are able to ferment a common set of sugars, which include glucose, sucrose and raffinose (Fig. S5, panel a). In contrast, fermentation capabilities for other sugars, such as lactose, galactose, maltose, melibiose, fructose, lactulose, maltodextrins, turanose, β-gentibiose and xylose, were shown to be variable for the majority of the strains tested (Fig. S5, panel a). Notably, we identified bifidobacterial taxa such as *B. cuniculi* displaying an ability to grow on a wide range of simple and complex carbohydrates (Fig. S5, panel a), thus suggesting metabolic expansion of its carbohydrate acquisition abilities perhaps to enhance competitiveness in one or more ecological niches. In contrast, other bifidobacterial species, such as *Bifidobacterium animalis* subsp. *animalis*, only utilize a relatively small number of the carbohydrates assayed here (Fig. S5, panel a), an observation which, together with the very limited number of predicted GHs/carbohydrate pathways encoded by this taxon (Fig. 1), underlines a rather high level of genetic adaptation to an ecological niche. Growth studies highlighted the existence of different carbon sources that are differentially utilized by bifidobacteria, in a manner consistent with our predictions. In this context growth experiments involving most members of the *B. asteroides* phylogenetic group showed that they do not exhibit any appreciable growth on glycogen, an observation which is consistent with *in silico* pathway predictions. Cultivation trials performed on plant-derived carbohydrates such as arabinose or xylose revealed that these sugars are utilized by a majority of the bifidobacteria tested except for those taxa that form the GHP/B cluster (Fig. 1), which are not predicted to encode enzymes of the L-arabinose degradation I and xylose degradation I pathways. The ubiquitous monosaccharide mannose that is found in both plant and animal glycans, is easily shunted into the glycolytic pathway via isomerization of mannose-6-phosphate to fructose-6-phosphate^30^. Growth on mannose is, however, not a wide-spread property among the tested bifidobacterial taxa, a finding that is consistent with genomic data (Fig. 1), which revealed a variable distribution of genes supporting bifidobacterial mannose utilization such as genes encoding predicted mannosidases and mannose-6-phosphate isomerases. Interestingly, when mannosidase-encoding genes were detected, they were in the large majority of cases shown to be located within a putative N-glycan (host-derived) degradation cluster. Lactose constitutes a typical glycan that specifically occurs in the mammalian diet, though normally limited to the early stages of life^31^. *In silico* analyses involving the genomes of 47 bifidobacterial taxa revealed a ubiquitous distribution in their chromosomes of genes encoding β-galactosidases. This finding suggests a rather wide-spread utilization of lactose as well as other galactose-containing glycans such as galactan, galacto-oligosaccharides, Human milk oligosaccharides (HMO) and mucin, which require particular β-galactosidases for their degradation, among members of the *Bifidobacterium* genus^20^,^32^. Growth experiments involving lactose showed that, except for *B. ruminantium* and *B. thermacidophilum* subsp*. thermacidophilum*, all other tested bifidobacteria are able to ferment this disaccharide. In contrast, *in silico* analyses suggested that L-rhamnose is rarely used by bifidobacteria due to the apparent lack of genes encoding L-rhamno-gamma-lactonase, L-rhamnoate dehydratase and 2-keto-3-deoxy-L-rhamnoate aldolase. Notably, fermentation profiles involving this carbon source confirmed that only *B. biavatii* is able to ferment L-rhamnose. Furthermore, we evaluated the abilities of members of the genus *Bifidobacterium* to utilize typical host glycans like mucin and HMO. Interestingly, in addition to the currently known bifidobacterial archetypes that can utilize these host-glycans (*B. bifidum* and *B. longum* subsp. *infantis*)^20^,^33^, we identified that *B. biavatii, B. crudilactis, B. kashiwanohense, Bifidobacterium stellenboschense* and *Bifidobacterium mongoliense* are all capable of HMO metabolism. Inspection of their genome sequences revealed the presence of genes predicted to encode sialidases, fucosidases, N-acetyl-β-hexosaminidases, endo-α-N-acetylgalactosaminidase and β-galactosidases, which have previously been shown to be crucial for the breakdown of these complex carbohydrates^20^,^33^. This further illustrates the broad catabolic abilities of bifidobacteria in general and in particular their specialization to utilize complex carbohydrates that are commonly found in the (infant) mammalian GIT.

In order to substantiate the notion that bifidobacterial genomes contain specific genes responsible for the utilization of key carbohydrates that are present in their ecological niches and correspond to their saccharolytic phenotype, we investigated the transcriptome for a representative bifidobacterial species for each of the seven bifidobacterial phylogenetic clusters described previously^10^ grown on (where possible) glucose, glycogen, lactose, xylose, rhamnose, mannose, trehalose or HMO as the sole carbon source (Fig. S5, panel b). RNAseq experiments allowed the identification of the transcriptomes for each bifidobacterial strain tested in cases where growth was obtained, revealing that the carbohydrate metabolism COG family [COG category (G)], is one of the most represented in the transcriptomes (Fig. S5, panel c). Manual inspection of the identified transcriptomes highlighted the existence of a large arsenal of genes encoding GHs and other enzymes that constitute parts of carbohydrate metabolic pathways, including suspected carbohydrate carrier systems that are expressed when bifidobacteria are cultivated on a carbohydrate (Fig. S6). In this context, we identified β-galactosidases of the GH2 and GH42 families that were shown to be expressed when bifidobacteria are grown on lactose, as well as MFS and ABC systems predicted to act as carriers for lactose and/or glucose and/or galactose (Fig. S6). Cultivation of bifidobacteria on mannose, rhamnose, trehalose or xylose resulted, in cases where growth was observed, in the transcription of genes encoding specific catabolic pathways such as D-mannose degradation, L-rhamnose degradation II, trehalose degradation I and xylose degradation I (Fig. S6), observations that are consistent with our *in silico* assignments. The transcriptomes of bifidobacteria grown on glycogen clearly showed that transcription of genes encompassing the glycogen degradation I pathway, predicted to be indispensable for glycogen to glucose breakdown, is switched on under these circumstances (Fig. S6). The only exceptions are represented by the transcriptomes of *Bifidobacterium pseudocatenulatum* and *B. stellenboschense* in which the genes predicted to specify amylomaltase and glucokinase enzymes are not expressed, suggesting the existence of an alternative pathway for conversion of the intermediate carbohydrate maltose into β-D-glucose-6-phosphate. Transcriptomic data recovered from growth of *B. biavatii* on the complex substrate HMO revealed up-regulation of a sizable number of different GH-encoding genes, such as those predicted to belong to GH1 (predicted β-D-fucosidases), GH2 (putative β-galactosidases), GH3 (putative β-N-acetylhexosaminidases), GH29 (α-L-fucosidases), GH30 (possible β-fucosidases), GH36 (likely α-N-acetylgalactosaminidases), GH42 (putative β-galactosidases), GH85 (endo-β-N-acetylglucosaminidases) and GH112 (predicted galacto-N-biose/lacto-N-biose phosphorylases) (Fig. S6). Other up-regulated GH-coding genes included those specifying members of GH13, GH32 and GH43 families, even though their predicted enzymatic activities do not appear to be directed against HMO.

### Mutualistic/commensal breakdown activities of bifidobacteria on carbohydrates

**Figure 3:**
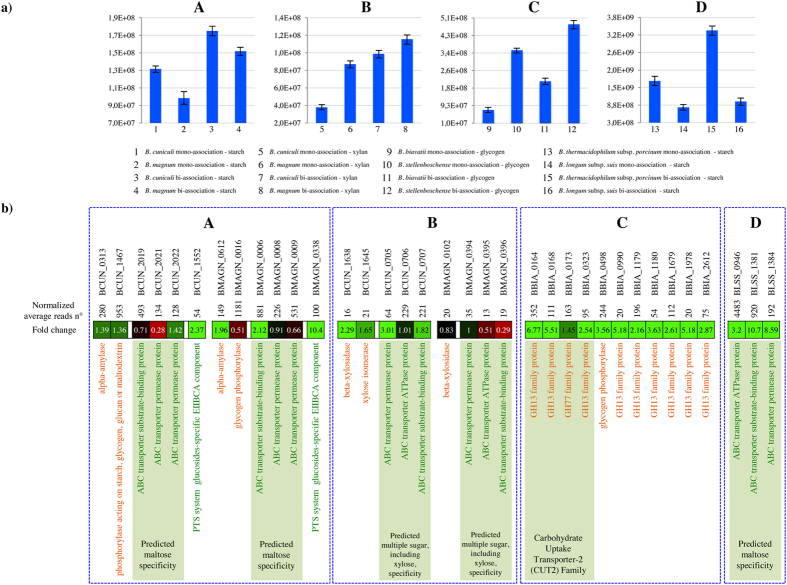
Evaluation of possible bifidobacterial cross-feeding by a transcriptomics approach. (Panel **a**) reports the abundance, observed through quantitative qRT-PCR, of eight bifidobacterial species cultivated in MRS supplemented with four different carbohydrates. These species were either grown on their own (mono-association) or in the presence of another bifidobacterial strain (bi-associations) sharing the same ecological niche. The five case studies analysed are named progressively with letters from A to D, corresponding to: *B. cuniculi* and *B. magnum* grown on starch (A), *B. cuniculi* and *B. magnum* grown on xylan (B), *B. biavatii* and *B. stellenboschense* grown on glycogen (C) and *B. thermacidophilum* subsp*. porcinum* and *B. longum* subsp*. suis* grown on starch (D). (Panel **b**) shows the transcriptional fold change of genes encoding enzymes in the breakdown of glycans observed in the five case studies, named progressively with letters from A to D. Functional annotation of enzymes are indicated in orange while the functional annotation of transporter encoding genes and the predicted glycan specificity is highlighted in green.

In gut ecosystems, bacteria can exploit mutualistic as well as commensal and competitive activities during metabolism of various carbon sources available in such an environment. Thus, we were interested to evaluate possible trophic relationships towards carbohydrates between bifidobacterial taxa found in various ecological niches. Specific bi-association of bifidobacterial taxa were selected based on their common ecological origin (e.g., human-, porcine-, or rabbit-gut). Their growth performances on carbon sources that are commonly available in their particular ecological niche were assayed during co-cultivation (bi-association) and compared to those achieved when the strains were grown separately (mono-association). Such trials suggested that when cultivated together these bifidobacterial taxa were taking an evident benefit as supported by the enhanced cell densities in the bi-associations compared to the mono-associations (Fig. 3). These growth-benefits were in a few cases evident for both strains such as in the case of the *B. magnum* and *B. cuniculi* combination when these strains were cultivated on xylan or on starch (Fig. 3). Such findings were further supported by the evaluation of transcriptomic changes, employing an RNAseq approach, observed for these bifidobacterial strains when cultivated together as compared to the situation where these strains were assayed separately. Notably, in bi-associations where a benefit was noticed in terms of cell numbers for both strains, such as for *B. magnum* and *B. cuniculi* cultivated on starch, complementarity in terms of the transcription of the genetic repertoire for the metabolism of this complex sugar was observed for these strains. In fact, under such conditions the alpha-glucoside phosphorylase-specifying genes, i.e. BMAGN_0016 and BCUN_1467, as well as amylase-encoding genes, i.e. BMAGN_0612 and BCUN_0313, of both *B. magnum* and *B. cuniculi* were shown to be transcribed (Fig. 3). Interestingly, while these latter enzymes are predicted to be intracellular, two genes encoding putative extracellular pullulanases (BCUN_0354 and BCUN_0356), were shown to be transcribed. Even though the detected transcription level for these genes was low, it may be that such enzymes allowed a partial de-branching of starch so as to allow internalization of the released degradation products into the bacterial cell for complete degradation. The resulting starch-degradation products such as maltose and alpha-glucosides are presumed to be internalized by means of ABC transporters (BMAGN_0006-BMAGN_0009 and BCUN_2019-BCUN_2022) and/or PTS systems (BMAGN_0338 and BCUN_1552), of which the corresponding genes were shown to exhibit increased transcription (2.37 fold and 10.42 fold with p < 0.001, respectively) relative to mono-association conditions (Fig. 3). Thus, on this substrate both strains appear to co-operate in order to achieve starch degradation thanks to the collective action of their extracellular amylases, perhaps resulting in the production of a larger amount of starch-derivatives compared to that achieved when the strains are cultivated separately on this substrate. In contrast, when these strains were co-cultivated on xylan, we only noticed an up-regulation of the gene encoding a beta-xylosidase (BCUN_1638) in *B. cuniculi*. Similarly, *B. cuniculi* exhibited a modest transcriptional upregulation of the genes specifying the putative uptake and degradation machinery for xylose (BCUN_0705-BCUN_0707 and BCUN_1645), whereas *B. magnum* showed no modulation in the beta-xylosidase-encoding gene, yet down-regulation of genes involved in xylose transport. The latter findings suggest that *B. magnum* modulates gene expression in order to support growth of *B. cuniculi* and allows this latter strain to participate in xylan degradation and harvesting of the deriving xylose. Since none of these enzymes are predicted to be extracellular, an alternative explanation for this behavior is partial degradation of xylan to xylo-oligosaccharides during media preparation. These degradation products can be the target for specific uptake transporters, allowing their complete breakdown inside the bacterial cells. Another scenario was noticed for the co-cultivation of *B. stellenboschense* and *B. biavatii* grown in the presence of glycogen, where only the latter strain seems to take advantage of the presence of the other strain (Fig. 3). In fact, under these circumstances *B. biavatii* substantially enhanced transcription of various genes encoding enzymes predicted to be involved in the hydrolysis of glycogen such as predicted glycosyl hydrolases of the GH13 and GH77 families, as well as a glycogen phosphorylase (Fig. 3). In contrast, *B. stellenboschense* did not reveal any transcriptional modulation of genes with predicted functions in glycogen utilization when the strains were co-cultivated (Fig. 3). Similarly, data concerning co-cultivation of *B. longum* subsp. *suis* and *B. thermacidophilum* subsp. *porcinum* on starch is also consistent with a cross-feeding scenario. In fact, under these growth conditions, qPCR assays revealed that cell numbers of *B. longum* subsp. *suis* are enhanced (≥2 fold) with respect to those noticed when this strain was cultivated on its own on this substrate. Furthermore, transcriptome analyses revealed a significant induction (≥8 fold, p < 0.001) of genes encoding a complete ABC system involved in the up-take of maltose, which is a starch-derived glycan, of *B. longum* subsp. *suis* when grown with *B. thermacidophilum* subsp. *porcinum* in the presence of starch as the sole carbon source (Fig. 3). In contrast, even though the *B. thermacidophilum* subsp. *porcinum* genome is predicted to encode a secreted GH13 family enzyme (BPORC_0608), the transcription of genes predicted to be involved in starch metabolism of *B. thermacidophilum* subsp. *porcinum* did not appear to be affected by the presence of *B. longum* subsp. *suis* (Fig. 3).

Overall, our findings suggest that bifidobacteria access carbohydrates that are commonly present in their ecologic niches employing trophic interactions that may vary from commensalism to mutualistic. Furthermore, these concerted breakdown activities may similarly exert positive effects on other members of the gut microbiota and thus promote an expansion of the gut glycobiome.

### Bifidobacteria and mammalian gut adaptation

**Figure 4:**
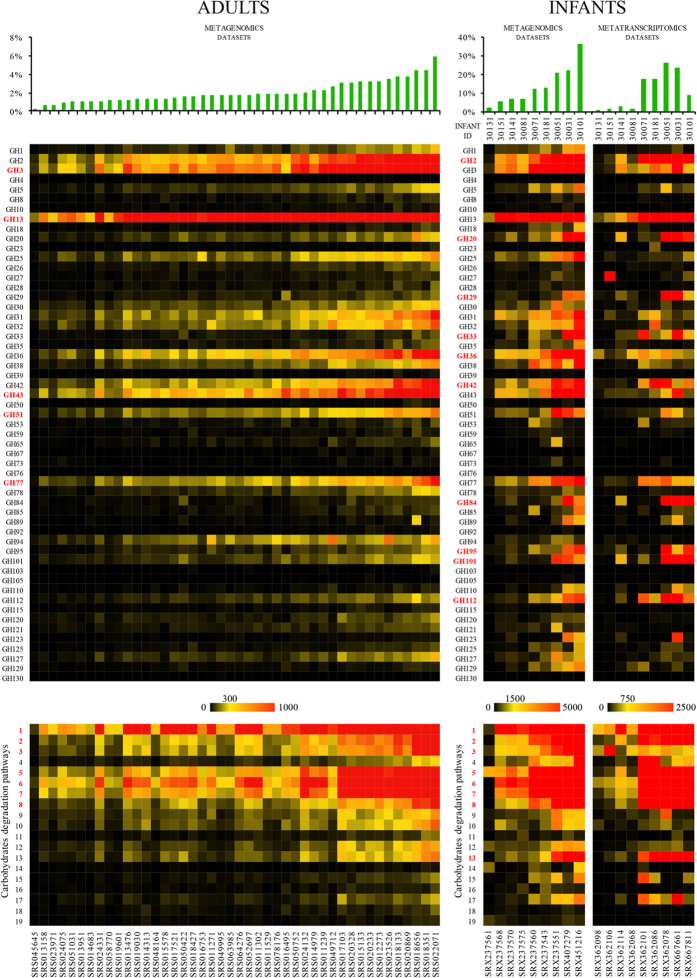
Data mining for bifidobacterial GH genes and bifidobacterial pathways for carbohydrate degradation in adult and infant fecal metagenome data sets and an infant fecal metatranscriptome data set. Bar plots above the heatmaps show the relative abundance of bifidobacteria in the analysed samples. Heatmaps in the upper part depict the coverage obtained by alignment of adult and infant fecal metagenomic data sets, or infant metatranscriptome data sets to predicted bifidobacterial GH-encoding genes. In order to compare results for datasets with different sizes, all coverage values were normalized as obtained from a 10 million read dataset. Heatmaps in the lower part of the image represent the coverage obtained by alignment of the same datasets to genes constituting the bifidobacterial pathways for carbohydrate degradation. Relevant GH genes and pathways involved in the metabolism of glycans are highlighted in red. Pathways designations are identical to those indicated in Fig. 1.

Bifidobacteria have predominantly been identified in the GIT of mammals^1^. However, their functional contribution to the microbiota residing in this body compartment has not been thoroughly investigated. We therefore looked for the presence of GH-encoding bifidobacterial DNA sequences among 44 out of 136 available gut metagenome data sets from healthy adults, enrolled in the Human Microbiome Project^19^ that had shown consistent presence of bifidobacteria based on MetaPhlAn profiling^34^, as well as in nine metagenomes and related metatranscriptomes of healthy infant gut microbiota, sequenced by the Broad Institute (NCBI bioproject ID 63661). The coverage of each gene included in the bifidobacterial pangenome was computed based on the metagenomic reads with >98% full-length identity. As displayed in Fig. 4, the average abundance of *Bifidobacterium* genes ranged from 0.2% to 35.9%, with a distinctly higher prevalence of bifidobacteria in the faecal metagenomes from infants. This is consistent with the catabolic potential for HMOs. Interestingly, among the most frequently represented bifidobacterial genes in the metagenomic data sets from adult, the presence of an extensive repertoire of GH-encoding genes, such as those specifying GH3, GH13, GH43, GH51 and GH77 (involved in the breakdown of complex plant carbohydrates), is noteworthy (Fig. 4). Such findings reinforce the notion that despite the relative paucity of bifidobacteria detected in the adult human gut, their functional contribution to the human gut microbiome may be important in terms of expanding the overall glycobiome of the large intestine, thereby affecting the overall gut physiology. Furthermore, in the metagenome datasets from infants the relatively high bifidobacterial abundance is also reflected by the prevalence of bifidobacterial GH-encoding genes, such as those specifying members of the GH2, GH20, GH29, GH33, GH36, GH42, GH84, GH95, GH101 and GH112 families, involved in the degradation of milk-related carbohydrates such as lactose and HMOs, as well as in mucin degradation (Fig. 4). Notably, GH-specifying genes encoding β-hexosaminidases, lacto-N-biosidases, galacto-N-biose/lacto-N-biose phosphorylases, α-L-fucosidases, sialidases, neuraminidases, N-acetyl β-glucosaminidases, hyaluronidases and endo-α-N-acetylgalactosaminidases are not widespread in adult metagenomes (Fig. 4). Availability of metatranscriptomes corresponding to the analysed infants’ metagenomes revealed pronounced transcription of the bifidobacterial GH gene predicted to be involved in milk, HMO and mucin degradation (Fig. 4), clearly supporting the key functional roles exploited by these GHs in the infant gut microbiota.

To further elucidate the functional contribution of bifidobacteria to the human microbiota we screened the metagenomic and metatranscriptomic datasets for the presence of genes encompassing carbohydrate degradation pathways that had been predicted to be present in the bifidobacterial pan-genome (Figs 1 and 4). Both the adult and infant metagenomes showed a high abundance of genes for the *Bifidobacterium* phosphoketolase-dependent, or so-called bif shunt, pathway, as expected, as well as genes involved in galactose degradation I, melibiose degradation, lactose degradation III, glycogen degradation I, glycogen degradation II and sucrose degradation IV pathways (Fig. 4), accompanied by presence of genes involved in starch degradation V, arabinose degradation I, xylan degradation and xylose degradation I pathways. The presence of such pathways illustrates their presumed importance for adaptation of bifidobacteria to the human gut environment. Interestingly, the infant’s metatranscriptomic datasets correspond to the presence of the bif shunt (as expected) and pathways for galactose, lactose, glycogen and sucrose degradation, while no or low transcriptional activity was detected that corresponded to catalytic pathways for the plant polysaccharides arabinose, xylan and xylose (Fig. 4), reflecting the paucity of these carbohydrates in an infant’s diet.

## Conclusions

Bifidobacteria may be considered as key representatives of the mammalian gut microbiota, especially during the first phase of their host’s life. However, very little is known about their genetic strategies to colonize and persist within the gut, and to get access to the nutrients available in this environment. The current study highlights the very extensive saccharolytic features displayed by members of the *Bifidobacterium* genus, revealing how these bacteria metabolize specific carbohydrates available in their particular ecological niche. Comparison of the glycobiome identified in the genus *Bifidobacterium* with those identified in other members of the human gut microbiota revealed their unique and important contribution in terms of GHs involved in the breakdown of complex plant carbohydrates such as arabinoxylan, galactan and starch. The impact of bifidobacteria in the breakdown of dietary carbohydrates is also crucial for the establishment and reinforcement of trophic relationships between members of the gut microbiota. In fact, both mutualistic as well as commensal interactions in the mammalian gut can be carbohydrate-driven^6^,^7^. Here, we have shown how a simple bifidobacterial community may co-operate between themselves as well as with other members of the gut microbiota in the utilization of specific glycans, commonly available in the mammalian gut, by means of cross-feeding activities so as to provide growth benefits to one or both members of such a community as well as with the other members of the gut microbiota. Such findings support the concept of the existence of a social intelligence of bifidobacterial communities in the harvesting and metabolism of glycans available in their ecological niches, which regulate the dynamics of the gut microbiota relationships. However, it is reasonable to expect that in much more complex microbial communities such as those identified in the mammalian gut, cross-feeding activities as exemplified by bifidobacteria also involve other members of the gut microbiota, perhaps generating an even larger beneficial effect. A survey of human gut metagenomic datasets, representing both adult and infant samples, revealed that, notwithstanding their relatively low abundance in adults, the functional contribution of bifidobacteria to the enzymatic arsenal directed at degradation of complex carbohydrates is relevant. In this context, the expansion of the GH repertoire dedicated to the metabolism of infant dietary sugars, such as HMOs, as well as mucin is noteworthy. Another clear sign of advanced bifidobacterial adaptation to its mammalian host is represented by the identification in a small number of bifidobacterial taxa which include *B. bifidum*, *B. longum* subsp. *infantis*^20^,^33^, as well as *B. biavatii, B. crudilactis, B. kashiwanohense, B. stellenboschense* and *B. mongoliense,* of metabolic repertoires involved in the breakdown of host-derived glycans such as HMOs and mucin.

## Materials and Methods

### Bacterial strains, growth conditions

All *Bifidobacterium* strains were cultivated in an anaerobic atmosphere (2.99% H_2_, 17.01% CO_2_ and 80% N_2_) in a chamber (Concept 400, Ruskin) on De Man-Rogosa-Sharp (MRS) broth (Scharlau Chemie, Barcelona, Spain) supplemented with 0.05% (w/v) L-cysteine hydrochloride and incubated at 37 °C. Cell growth on semi synthetic MRS medium supplemented with 1% (wt/vol) of a particular sugar was monitored by optical density at 600 nm using a plate reader (Biotek, Vermont, USA). The plate reader was run in discontinuous mode, with absorbance readings performed in 60 min intervals, and preceded by 30 sec shaking at medium speed. Cultures were grown in biologically independent triplicates and the resulting growth data were expressed as the mean of these replicates. Carbohydrates were purchased from Sigma (Milan, Italy) or Carbosynth (Berkshire, UK). All the carbohydrates were dissolved in water and then sterilized by filtration using 0.2 micron filter size and then added to autoclaved MRS with the exception of xylan (Poly(β-D-xylopyranose[1→4]) (Sigma, Aldrich) which was autoclaved with MRS.

In the case of HMO experiments, human milk samples were kindly provided by the Western Thrust Human Milk Bank (Irvinestown, Co. Fermanagh, Ireland). The isolation of oligosaccharides from pooled samples (n ≥ 3) was performed as described previously^35^. Briefly, samples were defatted by centrifugation at 4 °C (3850 × *g*, 20 min, Sorvall RC6 plus®). Caseins were precipitated at pH 4.6. After neutralization, large peptides were removed by ultrafiltration (5 kDa molecular-weight cut-off, Millipore® Helicon S10 Spiral Cartridge). The permeates were freeze-dried and stored at −80 °C until further processing. To remove lactose and residual peptides, the extracts were re-solubilized in MilliQ® water and applied to a Sephadex G-25 column (Pharmacia, Uppsala, Sweden; 92 × 2.6 cm). Elution was performed with deionized water (5 mL/min). Fractions were monitored for peptides according to Bradford^36^ and the lactose content was determined by HPAEC^35^. Fractions low in peptide- and lactose content were pooled and used for further characterization.

### Bifidobacterial and hosts phylogenetic reconstruction

The phylogeny of the 47 was reconstructed as described previously^9^,^10^. Phylogeny of the eukaryotic hosts was reconstructed through the SUPERFAMILY web tool^37^.

### Bifidobacterial transcriptomics identification through RNAseq assays

*B. biavatii, B. boum, B. stellenboschense, B. coryneforme, B. cuniculi, B. gallinarum* and *B. pseudocatenulatum* were selected as representatives for each of the seven phylogenetic groups. These strains were grown in MRS supplemented with glucose, glycogen, lactose, xylose, rhamnose, mannose, trehalose or HMO as a carbon source. In order to evaluate cross-feeding activities between two bifidobacterial strains, bacteria were co-cultivated on MRS supplemented with a particular glycan [RS2-resistant starch (Sigma, Aldrich), xylan (Poly(β-D-xylopyranose[1→4]) (Sigma, Aldrich) or glycogen (Sigma, Aldrich)], and growth was monitored by the evaluation of optical density at 600 nm followed by qRT-PCR using strain-specific primers (see below).

When growth was observed during logarithmic phase, cell pellets were resuspended in 1 ml of QUIAZOL (Quiagen, UK) and placed in a tube containing 0.8 g of glass beads (diameter, 106 μm; Sigma). The cells were lysed by shaking the mix on a BioSpec homogenizer at 4 °C for 2 min (maximum setting). The mixture was then centrifuged at 12,000 rpm for 15 min, and the upper phase containing the RNA-containing sample was recovered. The RNA sample was further purified by phenol extraction and ethanol precipitation according to an established method^38^. Quality and integrity of the RNA was checked by Agilent 2200 Tape Station Nucleic Acid System (Agilent Technologies, Palo Alto, Calif.). One hundred ng of total RNA was used as the starting input for RNA-Seq library preparation. Briefly, 100 ng of total RNA was treated with Ribo-Zero rRNA removal kit for Gram-positive bacteria (Epicentre, Madison, WI, U.S.A.) to remove rRNA according to the supplier’s instructions. The yield of rRNA depletion was checked by Agilent 2200 Tape Station Nucleic Acid System (Agilent Technologies, Palo Alto, Calif.). Then, rRNA-depleted RNA samples were fragmented using RNaseIII (Life Technologies, USA) followed by size evaluation using Experion (BioRad, UK). Whole transcriptome library was constructed using the Ion Total-RNA Seq Kit v2 (Life Technologies, USA). Barcoded libraries were quantified by qRT-PCR and each library template was amplified on Ion Sphere Particles using Ion One Touch 200 Template Kit v2 (Life Technologies, USA). Samples were loaded into 316 Chips and sequenced on the PGM (Life Technologies, USA). Sequencing reads were depleted of adapters, quality filtered (with overall quality, quality window and length filters) with FastqMcf ( https://code.google.com/p/ea-utils/) and aligned to the respective bifidobacterial reference genome through BWA^39^ with high stringency cut-offs (99% nucleotide identity) in order to accurately map reads of co-cultivation datasets on the correct genome. Counts of reads overlapping ORFs were performed using HTSeq ( http://www-huber.embl.de/users/anders/HTSeq/doc/overview.html) and analysis of the count data was performed using the R package DESeq2^40^. When the RNAseq analyses of mono-associations, DESeq2 output consists of fold induction values determined as the normalized number of transcripts identified for a given gene for bacterial cells cultivated on MRS containing a specific carbohydrate, relative to the number of identified transcripts for that same gene when the strain was grown on MRS containing glucose (reference condition). In case of RNAseq analysis of bi-associations, DESeq2 output consists of fold-induction values determined as the normalized number of transcripts identified for a given gene for bacterial cells cultivated in mono-association relative to the normalized number of identified transcripts for that same gene when the strain was cultivated in bi-association, using MRS supplemented with the same carbohydrate for both conditions. DESeq2 data normalization and differential gene expression analysis are based on the negative binomial distribution, which takes into consideration the overall transcript abundance identified for each analysed sample^40^.

### Evaluation of the cell density by qPCR

The co-cultivation with other bacteria was monitored by quantitative PCR (qPCR). The copy-number of a gene for a given strain used in the co-cultivation experiments was evaluated and compared to the growth rate of each individually cultivated microorganism. qPCR was performed using the CFX96 system (BioRad, CA, USA). Primers used in this study are listed in Table S2. Each PCR reaction mix contained the following: 7.5 μl 2× SYBR SuperMix Green (BioRad, CA, USA), 5 μl of DNA dilution, each of the forward and reverse primers at 0.5 μM and nuclease-free water was added to obtain a final volume of 15 μl. PCR products were detected with SYBR Green fluorescent dye and amplified according to the following protocol: one cycle of 95 °C for 3 minutes, followed by 39 cycles of 95 °C for 5 s and 60 °C for 20 s. Melting curve: 65 °C to 95 °C with increments of 0.5 °C/s. In each run, negative controls (no DNA) for each primer set were included. Standard curve was built using the CFX96 software (BioRad).

### Bifidobacterial gene survey of human gut metagenomic and metatranscriptomic datasets

We surveyed the presence of bifidobacterial genes into the microbial diversity of the healthy gut of 44 adults and nine infants. To this end we implemented a mapping-based pipeline to detect the presence and quantify the coverage of these gene categories annotated from the newly sequenced strains into the Illumina deep shotgun-sequenced metagenomic data sets derived from stool samples of the Human Microbiome Project (HMP)^19^ and into Illumina deep shotgun-sequenced metagenomic and metatranscriptomic data sets obtained from stool samples of nine healthy infants. The mapping was performed using BowTie2^41^ using multiple-hit mapping and “very-sensitive” policy. The mapping was post-processed with a custom script to retain those matches with at least 98% full-length identity with respect to at least one reference gene by threshold-ing the BowTie2 score at −12 for the 100 nt-long adults and infant datasets (−6 is the penalty for a high-quality mismatch). Genes covered by matching reads for less than 90% of their full length were discarded and the final gene-wise average coverage was computed using SAMtools^42^ and BEDtools^43^. In the final matrices, GHs genes and genes constituting carbohydrate-degrading pathways were collapsed into GH families and MetaCyc pathways. The estimation of the relative abundance of bifidobacteria (at the genus level) was performed with MetaPhlAn^34^.

### Data Deposition

The RNAseq data were deposited in SRA database under the following study accession numbers: PRJNA239567 and PRJNA277297.

## Additional Information

**How to cite this article**: Milani, C. *et al.* Bifidobacteria exhibit social behavior through carbohydrate resource sharing in the gut. *Sci. Rep.* **5**, 15782; doi: 10.1038/srep15782 (2015).

## Supplementary Material

Supplementary Information
