# Supplementary material for: Bifidobacteria exhibit social behavior through carbohydrate resource sharing in the gut
Source: Sci Rep. 2015 Oct 28;5:15782. doi: 10.1038/srep15782 (PMC4623478; doi:10.1038/srep15782)
Supplement: Supplementary Information [file srep15782-s1.pdf]

Supporting Information

Bifidobacteria exhibit social behavior through carbohydrate resource sharing in the gut

Christian Milani[1], Gabriele Andrea Lugli[1], Sabrina Duranti[1], Francesca Turroni[2], Leonardo Mancabelli[1], Chiara Ferrario[1], Marta Mangifesta[1], Arancha Hevia[3], Alice Viappiani[1], Matthias Scholz[4], Stefania Arioli[1], Borja Sanchez[3¶], Jonathan Lane[5], Doyle V Ward[6], Rita Hickey[5], Diego Mora[7], Nicola Segata[4], Abelardo Margolles[3], Douwe van Sinderen[2] and Marco Ventura[1]

Laboratory of Probiogenomics, Department of Life Sciences, University of Parma, Italy[1]; Alimentary Pharmabiotic Centre and Department of Microbiology, Bioscience Institute, National University of Ireland, Cork, Ireland[2]; Departamento de Microbiologia y Bioquimica de Productos Lacteos, IPLA – CSIC, Villaviciosa, Asturias, Spain[3]; Centre for Integrative Biology, University of Trento, Trento, Italy[4]; Teagasc Food Research Centre, Moorepark, Cork, Ireland[5]; Broad Institute, 415 Main St., Cambridge, USA[6]; Department of Food Environmental and Nutritional Sciences, University of Milan, Italy[7].

[¶]Current address: Department of Analytical Chemistry and Food Science, Faculty of Food Science and Technology. University of Vigo, Spain

Corresponding author. Mailing address for Marco Ventura Department of Life Sciences, University of Parma, Parco Area delle Scienze 11a, 43124 Parma, Italy. Phone: ++39-521-905666. Fax: ++39-521-905604. E-mail: marco.ventura@unipr.it

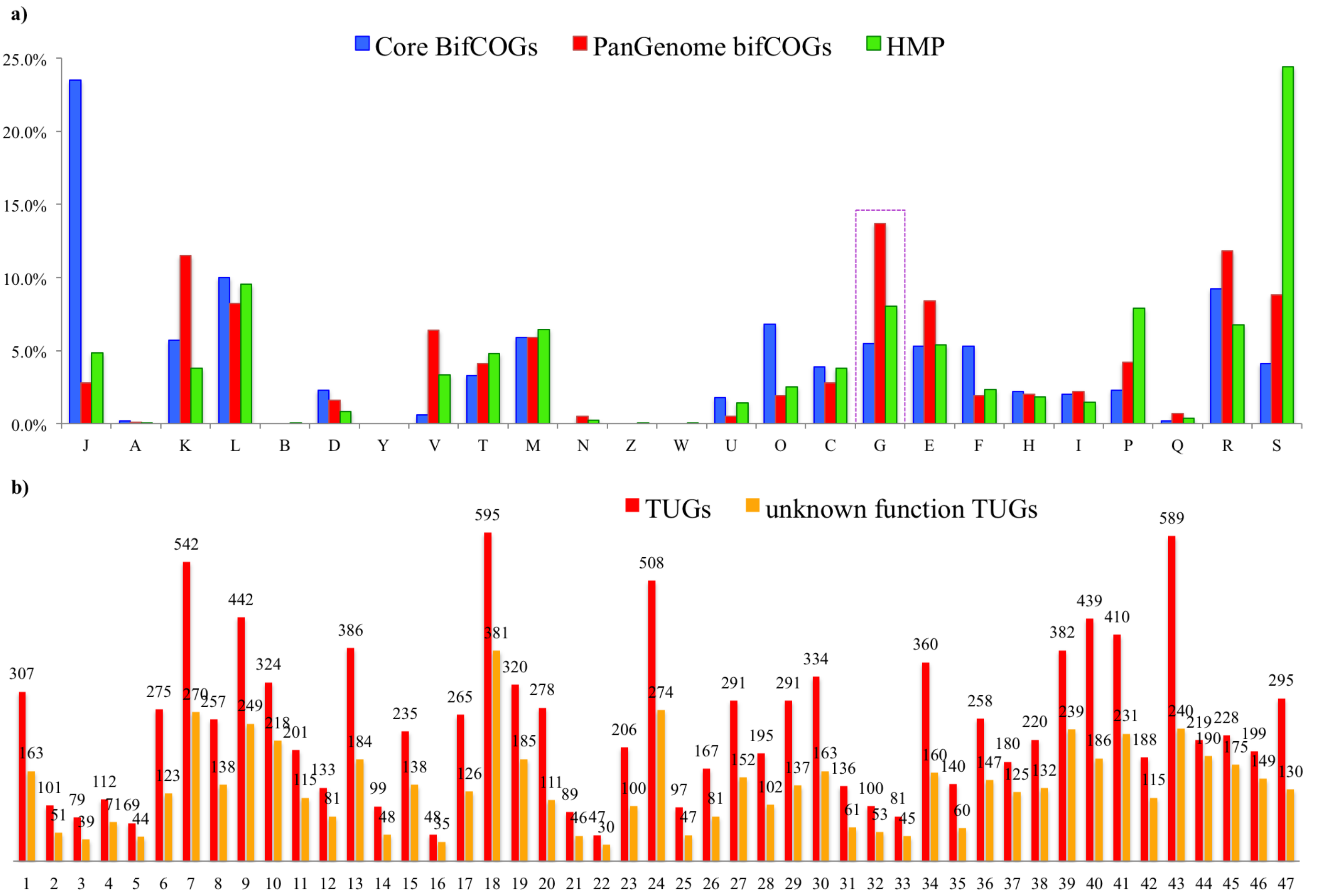
a)
Core BifCOGs
PanGenome bifCOGs
HMP
25.0%
20.0%
15.0%
10.0%
5.0%
0.0%
J A K L B D Y V T M N Z W U O C G E F H I P Q R S
b)
TUGs
unknown function TUGs
1 2 3 4 5 6 7 8 9 10 11 12 13 14 15 16 17 18 19 20 21 22 23 24 25 26 27 28 29 30 31 32 33 34 35 36 37 38 39 40 41 42 43 44 45 46 47

Fig. S1

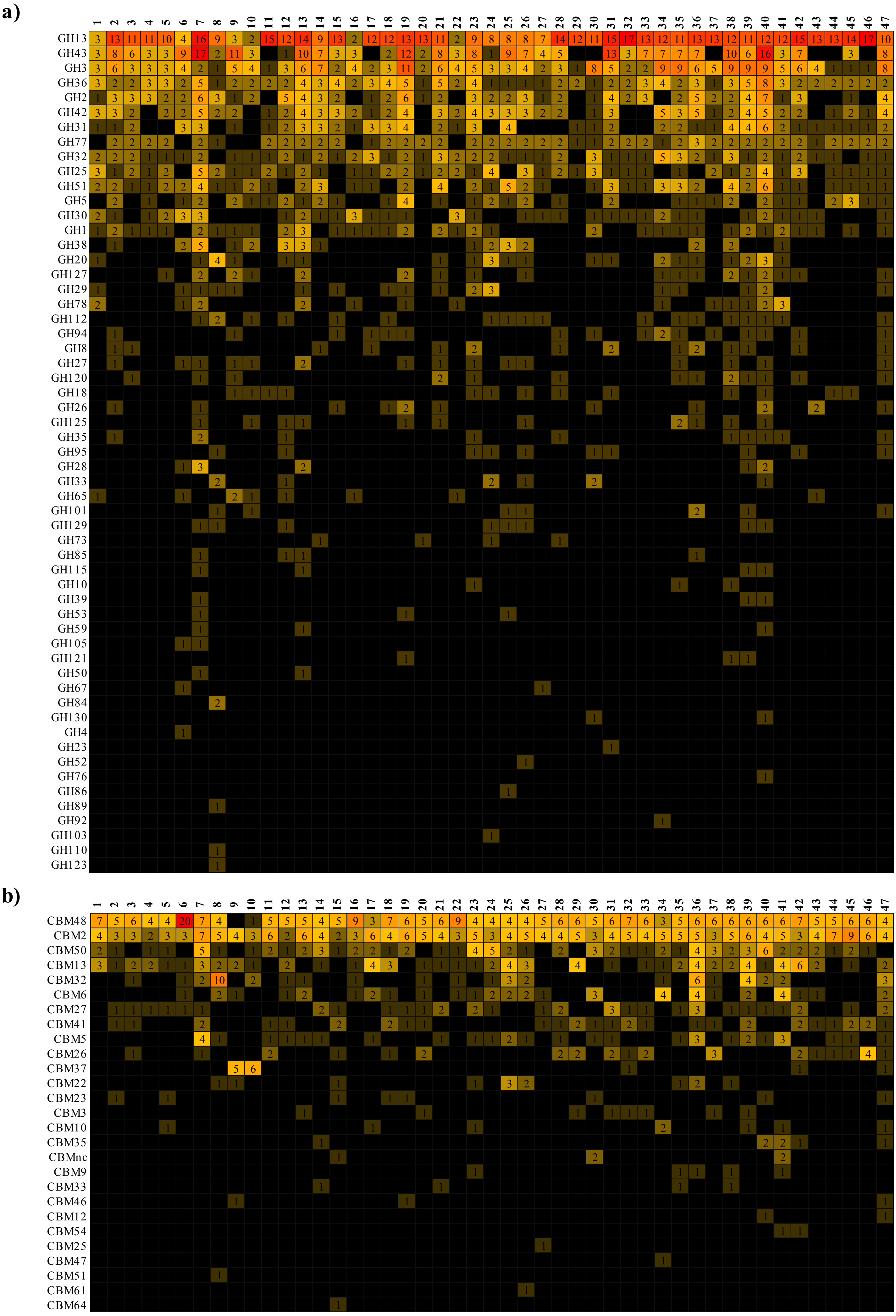
a)
1 2 3 4 5 6 7 8 9 10 11 12 13 14 15 16 17 18 19 20 21 22 23 24 25 26 27 28 29 30 31 32 33 34 35 36 37 38 39 40 41 42 43 44 45 46 47
GH13
GH43
GH3
GH36
GH2
GH42
GH31
GH77
GH32
GH25
GH51
GH5
GH30
GH1
GH38
GH20
GH127
GH29
GH78
GH112
GH94
GH8
GH27
GH120
GH18
GH26
GH125
GH35
GH95
GH28
GH33
GH65
GH101
GH129
GH73
GH85
GH115
GH10
GH39
GH53
GH59
GH105
GH121
GH50
GH67
GH84
GH130
GH4
GH23
GH52
GH76
GH86
GH89
GH92
GH103
GH110
GH123
b)
CBM48
CBM2
CBM50
CBM13
CBM32
CBM6
CBM27
CBM41
CBM5
CBM26
CBM37
CBM22
CBM23
CBM3
CBM10
CBM35
CBMnc
CBM9
CBM33
CBM46
CBM12
CBM54
CBM25
CBM47
CBM51
CBM61
CBM64

Fig. S2

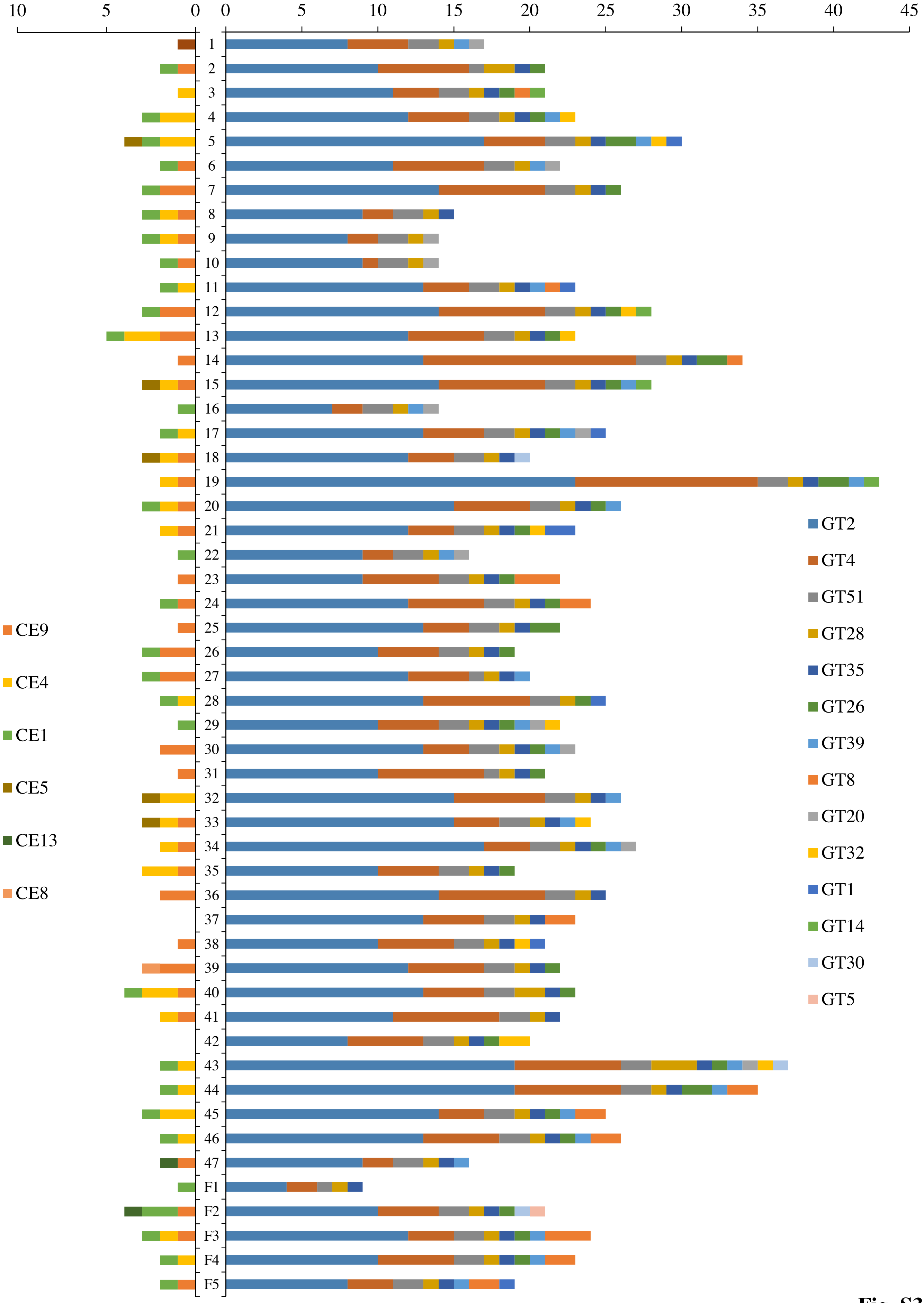

10
5
0
0
5
10
15
20
25
30
35
40
45
1
2
3
4
5
6
7
8
9
10
11
12
13
14
15
16
17
18
19
20
21
22
23
24
25
26
27
28
29
30
31
32
33
34
35
36
37
38
39
40
41
42
43
44
45
46
47
F1
F2
F3
F4
F5
CE9
CE4
CE1
CE5
CE13
CE8
GT2
GT4
GT51
GT28
GT35
GT26
GT39
GT8
GT20
GT32
GT1
GT14
GT30
GT5

Fig. S3

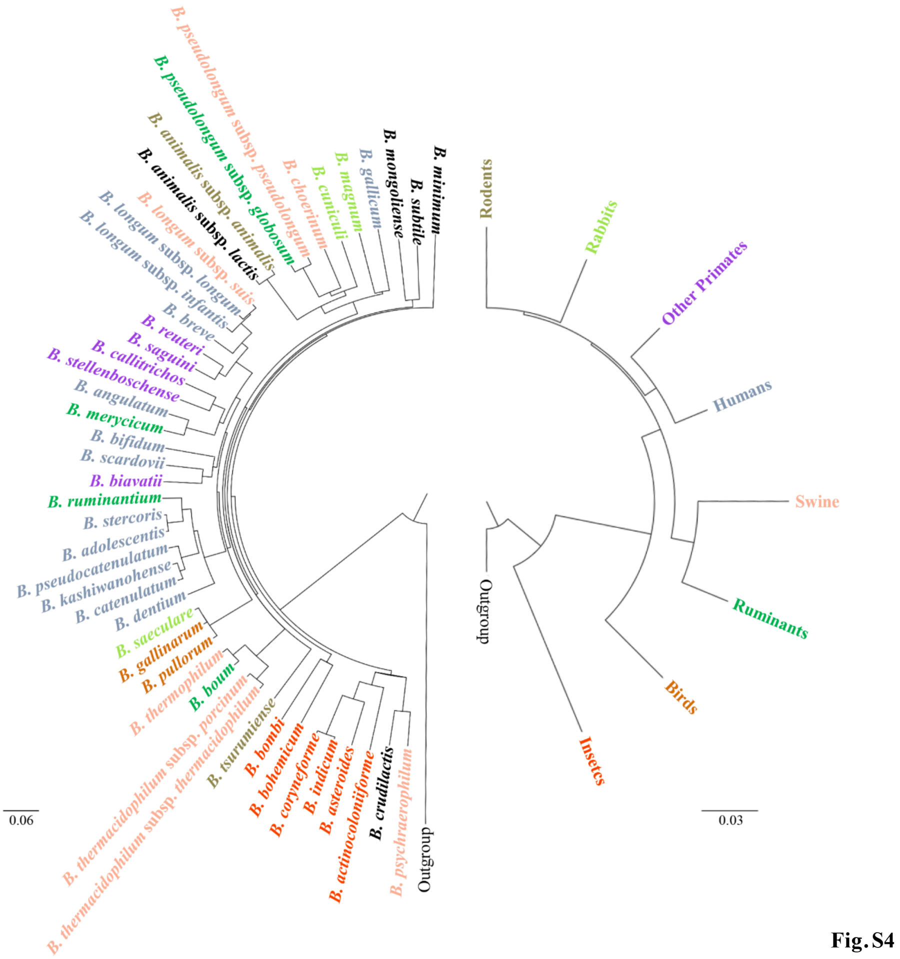
B. pseudolongum subsp. pseudolongum
B. pseudolongum subsp. globosum
B. animalis subsp. animalis
B. animalis subsp. lactis
B. longum subsp. suis
B. longum subsp. longum
B. longum subsp. infantis
B. choerinum
B. cuniculi
B. magnum
B. gallicum
B. mongoliense
B. subtile
B. minimum
B. breve
B. reuteri
B. saguini
B. callitrichos
B. stellenboschense
B. angulatum
B. merycicum
B. bifidum
B. scardovii
B. biavatii
B. ruminantium
B. stercoris
B. adolescentis
B. pseudocatenulatum
B. kashiwanohense
B. catenulatum
B. dentium
B. saeculare
B. gallinarum
B. pullorum
B. thermophilum
B. boum
B. thermacidophilum subsp. porcinum
B. thermacidophilum subsp. thermacidophilum
B. tsurumiense
B. bombi
B. bohemicum
B. coryneforme
B. indicum
B. asteroides
B. actinocoloniiforme
B. crudilactis
B. psychraerophilum
Outgroup
0.06
Rodents
Rabbits
Other Primates
Humans
Swine
Ruminants
Birds
Insects
Outgroup
0.03

Fig. S4

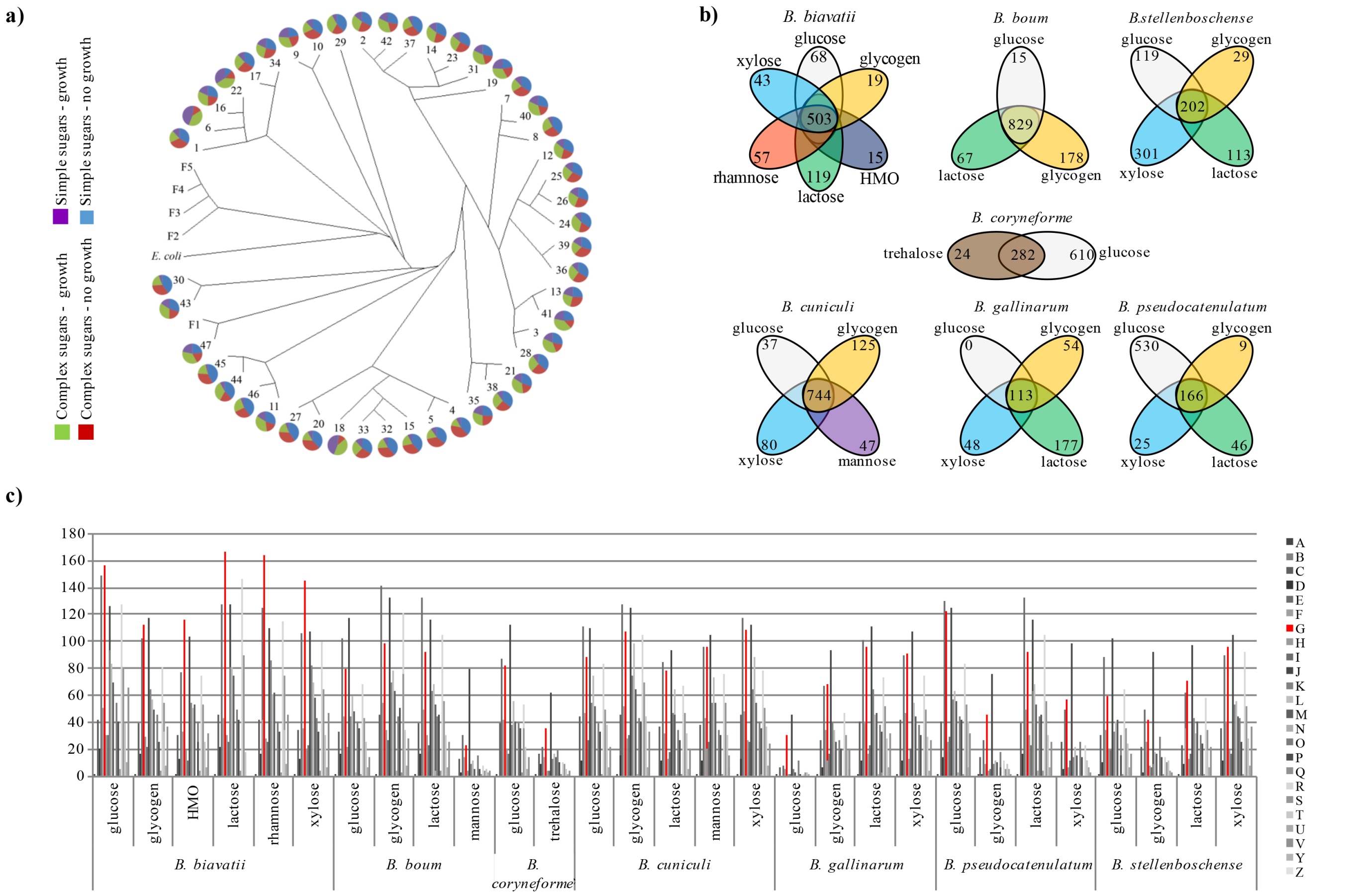
a)
Simple sugars - growth
Simple sugars - no growth
Complex sugars - growth
Complex sugars - no growth
E. coli
b)
B. biavatii
glucose
xylose
glycogen
rhamnose
HMO
lactose
68
43
19
503
57
15
119
B. boum
glucose
15
829
67
178
lactose
glycogen
B.stellenboschense
glucose
glycogen
119
29
202
301
113
xylose
lactose
B. coryneforme
trehalose
24
282
610
glucose
B. cuniculi
glucose
glycogen
37
125
744
80
47
xylose
mannose
B. gallinarum
glucose
glycogen
0
54
113
48
177
xylose
lactose
B. pseudocatenulatum
glucose
glycogen
530
9
166
25
46
xylose
lactose
c)
180
160
140
120
100
80
60
40
20
0
glucose
glycogen
HMO
lactose
rhamnose
xylose
mannose
trehalose
B. biavatii
B. boum
B. coryneforme
B. cuniculi
B. gallinarum
B. pseudocatenulatum
B. stellenboschense
A
B
C
D
E
F
G
H
I
J
K
L
M
N
O
P
Q
R
S
T
U
V
Y
Z

Fig. S5

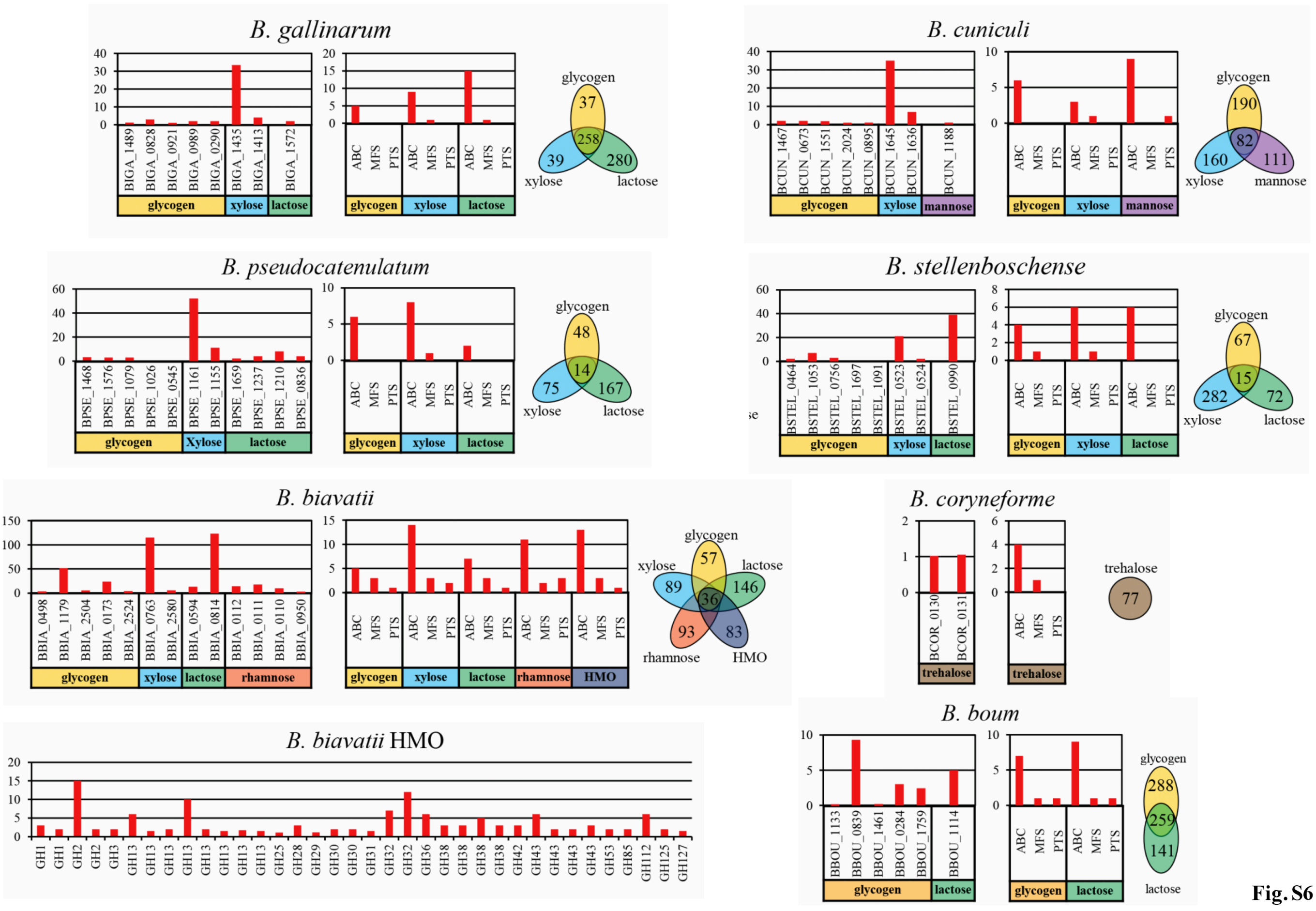

B. gallinarum
BIGA_1489
BIGA_0828
BIGA_0921
BIGA_0989
BIGA_0290
BIGA_1435
BIGA_1413
BIGA_1572
glycogen
xylose
lactose
ABC
MFS
PTS
glycogen
37
258
39
280
xylose
lactose
B. cuniculi
BCUN_1467
BCUN_0673
BCUN_1551
BCUN_2024
BCUN_0895
BCUN_1645
BCUN_1636
BCUN_1188
glycogen
xylose
mannose
190
82
160
111
B. pseudocatenulatum
BPSE_1468
BPSE_1576
BPSE_1079
BPSE_1026
BPSE_0545
BPSE_1161
BPSE_1155
BPSE_1659
BPSE_1237
BPSE_1210
BPSE_0836
Xylose
48
14
75
167
B. stellenboschense
BSTEL_0464
BSTEL_1053
BSTEL_0756
BSTEL_1697
BSTEL_1091
BSTEL_0523
BSTEL_0524
BSTEL_0990
67
15
282
72
B. biavatii
BBIA_0498
BBIA_1179
BBIA_2504
BBIA_0173
BBIA_2524
BBIA_0763
BBIA_2580
BBIA_0594
BBIA_0814
BBIA_0112
BBIA_0111
BBIA_0110
BBIA_0950
rhamnose
HMO
57
146
89
36
93
83
B. coryneforme
BCOR_0130
BCOR_0131
trehalose
77
B. biavatii HMO
GH1
GH1
GH2
GH2
GH3
GH13
GH13
GH13
GH13
GH13
GH13
GH13
GH13
GH25
GH28
GH29
GH30
GH30
GH31
GH32
GH32
GH36
GH38
GH38
GH38
GH38
GH42
GH43
GH43
GH43
GH43
GH53
GH85
GH112
GH125
GH127
B. boum
BBOU_1133
BBOU_0839
BBOU_1461
BBOU_0284
BBOU_1759
BBOU_1114
288
259
141

Fig. S6

Table S1. General features of the *Bifidobacterium* genomes.

Number	*Bifidobacterium* Strains	Genome Status	Genome Size	Isolation	Accession Number
1	*B. actinocoloniiforme* DSM 22766	Draft (4)	1823388	Bumblebee digestive tract	JGYK00000000
2	*B. adolescentis* ATCC 15703	Complete	2089645	Intestine of adult	AP009256.1
3	*B. angulatum* LMG 11039	Draft (6)	2003806	Human faeces	JGYL00000000
4	*B. animalis* subsp. *animalis* LMG 10508	Draft (13)	1915007	Rat feces	JGYM00000000
5	*B. animalis* subsp. *lactis* DSM 10140	Complete	1938606	Fermented milk	CP001606.1
6	*B. asteroides* LMG 10735 (PRL2011)	Complete	2167304	Honeybee hindgut	CP003325.1
7	*B. biavatii* DSM 23969	Draft (56)	3252147	Feces of tamarin	JGYN00000000
8	*B. bifidum* LMG 11041	Draft (2)	2208468	Brest-feed Infant faeces	JGYO00000000
9	*B. bohemicum* DSM 22767	Draft (5)	2052470	Bumblebee digestive tract	JGYP00000000
10	*B. bombi* DSM 19703	Draft (4)	1895239	Bumblebee digestive tract	ATLK00000000
11	*B. boum* LMG 10736	Draft (18)	2171356	Bovine rumen	JGYQ00000000
12	*B. breve* LMG 13208	Draft (31)	2263780	Infant intestine	JGYR00000000
13	*B. callitrichos* DSM 23973	Draft (33)	2887313	Feces of common marmoset	JGYS00000000
14	*B. catenulatum* LMG 11043	Draft (11)	2082756	Adult intestine	JGYT00000000
15	*B. choerinum* LMG 10510	Draft (20)	2096123	Piglet faeces	JGYU00000000
16	*B. coryneforme* LMG 18911	Complete	1755151	Honeybee hindgut	CP007287
17	*B. crudilactis* LMG 23609	Draft (6)	2362816	Raw cow milk	JHAL00000000
18	*B. cuniculi* LMG 10738	Draft (41)	2531592	Rabbit faeces	JGYV00000000
19	*B. dentium* LMG 11045 (Bd1)	Complete	2636367	Oral cavity	CP001750.1
20	*B. gallicum* LMG 11596	Draft (12)	2004594	Adult intestine	JGYW00000000
21	*B. gallinarum* LMG 11586	Draft (10)	2160836	Chicken caecum	JGYX00000000
22	*B. indicum* LMG 11587	Complete	1734546	Insect	CP006018
23	*B. kashiwanohense* DSM 21854	Draft (30)	2307960	Infant feaces	JGYY00000000
24	*B. longum* subsp. *infantis* ATCC 15697	Complete	2832748	Intestine of infant	AP010889.1
25	*B. longum* subsp. *longum* LMG 13197	Draft (8)	2384703	Adult intestine	JGYZ00000000
26	*B. longum* subsp. *suis* LMG 21814	Draft (36)	2335832	Pig faeces	JGZA00000000
27	*B. magnum* LMG 11591	Draft (13)	1822476	Rabbit faeces	JGZB00000000
28	*B. merycicum* LMG 11341	Draft (16)	2280236	Bovine rumen	JGZC00000000
29	*B. minimum* LMG 11592	Draft (18)	1892860	Sewage	JGZD00000000
30	*B. mongoliense* DSM 21395	Draft (43)	2170490	Fermented mare's milk	JGZE00000000
31	*B. pseudocatenulatum* LMG 10505	Draft (10)	2283767	Infant faeces	JGZF00000000
32	*B. pseudolongum* subsp. *globosum* LMG 11569	Draft (26)	1935255	Bovine rumen	JGZG00000000
33	*B. pseudolongum* subsp. *pseudolongum* LMG 11571	Draft (11)	1898684	Swine faeces	JGZH00000000
34	*B. psychraerophilum* LMG 21775	Draft (11)	2615078	Pig caecum	JGZI00000000
35	*B. pullorum* LMG 21816	Draft (11)	2153559	Chicken faeces	JGZJ00000000
36	*B. reuteri* DSM 23975	Draft (28)	2847572	Feces of common marmoset	JGZK00000000
37	*B. ruminantium* LMG 21811	Draft (23)	2249807	Bovine rumen	JGZL00000000
38	*B. saeculare* LMG 14934	Draft (14)	2263283	Rabbit faeces	JGZM00000000
39	*B. saguini* DSM 23967	Draft (33)	2787036	Feces of tamarin	JGZN00000000
40	*B. scardovii* LMG 21589	Draft (34)	3141793	Blood	JGZO00000000
41	*B. stellenboschense* DSM 23968	Draft (40)	2812864	Feces of tamarin	JGZP00000000
42	*B. stercoris* DSM 24849	Draft (15)	2304613	Adult feaces	JGZQ00000000
43	*B. subtile* LMG 11597	Draft (27)	2790088	Sewage	JGZR00000000
44	*B. thermacidophilum* subsp. *porcinum* LMG 21689	Draft (3)	2079368	Piglet faeces	JGZS00000000
45	*B. thermacidophilum* subsp. *thermacidophilum* LMG 21395	Draft (8)	2233072	Anaerobic digester	JGZT00000000
46	*B. thermophilum* JCM 1207	Draft (12)	2099496	Swine feaces	JGZV00000000
47	*B. tsurumiense* JCM 13495	Draft (25)	2164426	Hamster dental plaque	JGZU00000000

Table S2. qPCR primers used in this study.

Primer	Sequence (5'-3')	Targeted ORF/gene	Organism
Bbia-fw	GGATATGTTGTTCGTATGGGA	BBIA_0409	*B. biavatii* DSM 23969
Bbia-rv	GACTCGCTGTGTATGTGCTTC		
Bste-fw	ATACGGCAAGATCGTCAACAT	BSTEL_1950	*B. stellenboschense* DSM 23968
Bste-rv	GATTGACCTTGTTGTAGGCTT		
Blosu-fw	ACACCTTCCAATCTACTGCAT	BLSS_1597	*B. longum* subsp. *suis* LMG 21814
Blosu-rv	CATCACTGTCTACGAAACCCA		
Btp-fw	ACACCACGTATAGTCTCGGC	BPORC_0092	*B. thermoacidophilum* subsp. *porcinum* LMG 21689
Btp-rv	GCTTTTGGTTTTCTCCCGGT		
Bcun-fw	GTGATGTACCTGGTAGAAGGAA	BCUN_0008	*B. cuniculi* LMG 10738
Bcun-rv	TGTGCCGTATGTGGTGAGATAT		
Bmag-fw	CAATGCGAACTATGCGGACA	BMAGN_1584	*B. magnum* LMG 11591
Bmag-rv	AAATCAGCGCGAGTCCATTC		

Supplementary figures.

Figure S1. COG functional annotation and Truly Unique Genes (TUG) of the *Bifidobacterium* genus. Panel a shows the COG functional classification of the core BifCOGs in blue, pangenome BifCOGs in red, and average abundance in samples from the Human Microbiome Project in green. Panel b displays in red the TUG number in each bifidobacterial genome and in orange the number of TUGs with unknown function, representing 54.1 % of the total TUG pool. The number of TUG with predicted or unknown function is reported above each column in the graph. COG functional families are abbreviated as follow: [J] Translation, ribosomal structure and biogenesis, [A] RNA processing and modification, [K] Transcription, [L] Replication, recombination and repair, [B] Chromatin structure and dynamics, [D] Cell cycle control, cell division, chromosome partitioning, [Y] Nuclear structure, [V] Defense mechanisms, [T] Signal transduction mechanisms, [M] Cell wall/membrane/envelope biogenesis, [N] Cell motility, [Z] Cytoskeleton, [W] Extracellular structures, [U] Intracellular trafficking, secretion, and vesicular transport, [O] Posttranslational modification, protein turnover, chaperones, [C] Energy production and conversion, [G] Carbohydrate transport and metabolism, [E] Amino acid transport and metabolism, [F] Nucleotide transport and metabolism, [H] Coenzyme transport and metabolism, [I] Lipid transport and metabolism, [P] Inorganic ion transport and metabolism, [Q] Secondary metabolites biosynthesis, transport and catabolism, [R] General function prediction only, [S] Function unknown.

Figure S2. Prediction of the glycosyl hydrolases (GH) and carbohydrate-binding modules (CBM) in the *Bifidobacterium* genus. Panels a and b show, respectively, the GH and CBM families profile of all analysed bifidobacterial species, based on the CAZy database. Each bifidobacterial genome analysed is numbered according to the numbering of the species displayed in Supplementary Table 1.

Figure S3. Prediction of the glycosyl transferases (GT) and carbohydrate esterases (CE) in the *Bifidobacterium* genus. Left and right bar plots show respectively the CE and GT families profile of all the bifidobacterial species, based on the CAZy database. Each bifidobacterial genome analysed is numbered according to the numbering of the species displayed in Supplementary Table 1. Additional members of the *Bifidobacteriaceae* family (other than those belonging to the genus *Bifidobacterium*) are as follows: F1 *Gardnerella vaginalis*, F2 *Metascardovia criceti*, F3 *Parascardovia denticolens*, F4 *Scardovia inopinata* and F5 *Scardovia wiggsiae*.

Figure S4. Comparison between phylogenetic evolution of bifidobacterial species and their eukaryotic hosts. The tree on the left depicts the evolutionary development of the 47 analyzed members of the genus *Bifididobacterium*, while the tree on the right illustrates the phylogeny of their eukaryotic hosts. Colors are used to correlate each bifidobacterial species to their respective host.

Figure S5. Whole-genus carbohydrate utilization survey and glycan transcriptomic analyses of representative bifidobacterial species from the seven *Bifidobacterium* phylogenetic groups. Panel a shows a phylogenetic core-supertree of the *Bifidobacteriaceae* family. Each terminal node is a cake diagram describing the number of simple and complex carbohydrates that each tested bifidobacterial strain is able to utilize of a total of 23 simple carbohydrates and 27 complex sugars. Each bifidobacterial genome analysed is numbered according to the numbering of the species displayed in Supplementary Table 1. Remaining members of the *Bifidobacteriaceae* family are named as follows: F1 *Gardnerella vaginalis*, F2 *Metascardovia criceti*, F3 *Parascardovia denticolens*, F4 *Scardovia inopinata* and F5 *Scardovia wiggsiae*. Panel b shows a Venn diagram representation of the observed transcriptome of seven bifidobacterial taxa, chosen as representatives of the seven phylogenetic groups, grown on a total of eight simple or complex carbohydrates. Panel c consists of a bar plot representing the COG annotation of the observed transcriptomes. Highlighted in red is the carbohydrate metabolism and transport (G) COG class.

Figure S6. Expression modulation of genes involved in carbohydrate transport and metabolism possessed by representative bifidobacterial species from the seven *Bifidobacterium* phylogenetic groups. Differential expression are reported comparing normalized read counts for the different conditions versus growth on glucose. Venn diagrams depict the number of differentially transcribed (>2-fold increased transcription) genes in the different growth conditions as well as actively transcribed genes shared by all conditions. Bar plots represent fold transcriptional induction of genes involved in degradation pathways and the number of transporter-encoding genes overexpressed (>2-fold increased transcription). Glycogen degradation I is constituted by glycogen phosphorylase, glycogen debranching protein, phosphoglucomutase, amylomaltase, and glucokinase, xylose degradation I is constituted by xylose isomerase and xylulokinase, lactose degradation is constituted by β-galactosidases, L-rhamnose degradation II is constituted by L-rhamnofuranose 1-dehydrogenase, L-rhamnoate dehydratase, 2-keto-3-deoxy-L-rhamnoate aldolase and L-lactaldehyde dehydrogenase, while trehalose degradation I is constituted by trehalose phosphatase and α,α-phosphotrehalase.
